# Do biomedical researchers differ in their perceptions of plagiarism across Europe? Findings from an online survey among leading universities

**DOI:** 10.1186/s12910-022-00818-4

**Published:** 2022-08-08

**Authors:** Nannan Yi, Benoit Nemery, Kris Dierickx

**Affiliations:** 1 Department of Medical Humanities, School of Humanities, Southeast University, Nanjing, 211189 China; 2 Centre for Environment and Health, Department of Public Health and Primary Care, KU Leuven, Leuven, Belgium; 3 Centre for Biomedical Ethics and Law, Department of Public Health and Primary Care, KU Leuven, Leuven, Belgium

**Keywords:** Plagiarism, Perceptions, Biomedical researchers, Europe

## Abstract

**Background:**

Existing research on perceptions of plagiarism and cultural influences mainly focuses on comparisons between the Western World and the Eastern World. However, possible differences within the Western World have hardly been assessed, especially among biomedical academics. The authors compared perceptions of plagiarism among European biomedical researchers who participated in an online survey.

**Methods:**

The present work is based on the data collected in a previous online survey done in 2018 among biomedical researchers working in leading European and Chinese universities. Respondents based in Europe were grouped into three geographical regions (northern Europe, southern Europe and northwestern Europe) and their responses were analyzed using logistic regression analysis with adjustments for demographic factors.

**Results:**

Data were available from 810 respondents (265 northern Europe, 101 southern Europe, 444 northwestern Europe). In addition to their generally similar responses, different perceptions of plagiarism were observed among respondents in the three European regions. In summary, among the three European regions, Nordic respondents identified the most types of practices as plagiarism. Compared to the southern respondents, Nordic and northwestern respondents were more likely to consider less evident practices as plagiarism, such as Rephrasing another person’s work without crediting the source [aOR_N|S_ 1.99 (95%CI 1.08;3.67), aOR_S|NW_ 0.50 (95%CI 0.28;0.91)] and With permission from the original author, using another’s text without crediting the source [aOR_N|S_ 3.16 (95%CI 1.90;5.25), aOR_S|NW_ 0.26 (95%CI 0.16;0.42)]. In contrast, the southern respondents were the most inclined to recognize recycling of one’s previously rejected research proposal as plagiarism.

**Conclusions:**

In spite of a generally similar response pattern, the present study indicates different perceptions of plagiarism among European biomedical researchers. These intra-European differences should be considered when addressing plagiarism.

**Supplementary Information:**

The online version contains supplementary material available at 10.1186/s12910-022-00818-4.

## Introduction

Plagiarism is considered one of the most serious breaches of research integrity, on a par with data fabrication and falsification [1–3]. In contrast to data manipulation, however, the understanding of plagiarism is not always obvious and, moreover, plagiarism is thought to be influenced by culture, education, and other factors [4–6].

Plagiarism has a substantial negative impact on the scientific community [7]. It has been claimed that the Western world and other cultures (mostly Asia) differ in their understandings of plagiarism because of “cultural” and educational differences [8–11]. Such culturally determined differences in perceptions of plagiarism, if any, could have a great impact, especially in view of the growth of research collaborations across cultures. However, only limited evidence from empirical research is available to support that scientific professionals from different cultures diverge in their perceptions of plagiarism [9, 12, 13].

To provide more solid empirical data to fill this knowledge gap, we conducted, in 2018, an online survey about perceptions of plagiarism definition and collected responses from more than 1,000 biomedical researchers based in Europe and China. We initially focused on possible differences between European and Chinese researchers and, in an earlier publication [14], we reported that both groups had a generally good understanding of obvious plagiarism with only slight differences appearing between the perceptions of European and Chinese respondents.

In discussions about cultural differences, the “Western world” is often seen as a single homogeneous entity, ignoring the existence of social and cultural disparities across Europe [15–17]. Yet, it is conceivable that plagiarism is perceived differently within Europe. This led us to further analyze the responses obtained in our survey to determine similarities and dissimilarities in perceptions of plagiarism definition within biomedical researchers working in different countries within Europe.

## Methods

The current analysis is based on a subset of replies to an anonymous online survey in which a questionnaire was sent in 2018 to European and Chinese biomedical researchers, as described in a previous publication [14]. The replies received from biomedical researchers working in Europe are the focus of the present analysis.

### Survey instrument

The survey instrument was a self-designed questionnaire (see Additional file 1) based on the TURNITIN definition of plagiarism [18] and our research team's previous work [19, 20]. It was elaborated using a procedure similar to that used by Liao et al. [21] and finalized after a series of modifications based on feedback from experts and international researchers, as detailed in our previous paper [14].

The self-administered questionnaire (in English) consisted of three parts, beginning with questions to obtain demographic data, such as age, gender, academic position, and overseas research experience. The following section (Sect. 1) enquired about respondents' general opinions on plagiarism, such as ﻿ factors they perceived to determine whether a practice constitutes plagiarism. The final section (Sect. 2) examined respondents’ understanding of plagiarism, listing seven groups of practices and asking respondents to indicate which ones they thought were plagiarism.

### Selection and invitation of respondents

Our initial survey aimed to investigate biomedical researchers (researchers active in medicine, pharmaceutical sciences, and life sciences) from leading research universities in Europe and China. In total, we selected 46 universities, including 13 universities from the League of European Research Universities [22, 23] (those with medical schools, in consideration of the regional spread of universities) and 33 universities (those with biomedical schools) from China's Class A Universities of the Double First Class University (see Additional file 2) [24, 25].

﻿The first author NY manually collected the e-mail addresses of all target researchers (professors, associate professors, assistant professors, and postdoctoral researchers) whose email addresses were available on the university websites. E-mails with survey invitations (see Additional file 3) were sent to all of the target researchers (target researchers at KU Leuven were invited by the university, while researchers at the other universities were invited by the first author NY) in groups by university, but the names of the receivers were obscured, ensuring participant anonymity, followed by reminders [14]. In total, emails were dispatched to 14,757 researchers in Europe. All replies were gathered from March to July 2018. More details are available in the previous publication [14].

In our survey, we asked the country in which the researcher worked. For the present analysis we defined a priori three regions of interest based on the respondent’s workplace: Nordic countries (Denmark, Finland, and Sweden), Northwestern European countries (Belgium, France, Germany, the Netherlands, Switzerland, and the UK), and Southern European countries (Italy and Spain). Only those whose country of work fell inside one of the three regions were considered for further analysis. The three regions—Nordic countries, Northwestern European countries and Southern European countries—would be abbreviated to N, NW and S, respectively.

### Ethics approval

The study was approved by the Social and Societal Ethics Committee of the KU Leuven (dossier G- 2017 08 885).

The informed consent was obtained from all the participants in this study.

### Statistical methodology

The percentage of respondents choosing an option or answering yes to a question was computed (the number of respondents choosing the option/the total number of valid responses × 100) and displayed in tables and figures. For continuous variables, means and standard deviations were calculated and displayed.

The Chi square test was used to compare responses from the three regions for binary and categorical variables [age (in 10-year categories), gender, mother tongue (English or not), current academic position, PhD degree, year of obtaining PhD degree (in 10-year categories), and international research experience]. The Mann–Whitney U test was used to compare responses from the three regions for continuous variables (age). For binary and ordinal variables, logistic regression models and proportional odds models were used to compare respondents in the three regions (by pairwise comparisons of each two regions), adjusting for age, mother tongue (English or not), current academic position and PhD degree. The adjusted odds ratios (aORs) were calculated and their 95% confidence intervals (CIs) were reported. Some aOR values with subscripts, such as aOR_N|S_, aOR_N|NW_ and aOR_S|NW_ mean that the aOR value of N, N and S was calculated with the region S, NW, NW as the reference, respectively.

The null hypothesis was that the proportions of responses to the questions would not differ significantly between the three regions. When the two-tailed P value was less than 0.05, the null hypothesis was rejected.

SAS 9.4 was used to analyze the data.

This study's reporting adheres to STROBE statement [26].

## Results

With a response rate of 5.6%, we received 826 valid responses from the European respondents. We included 810 responses for further analysis based on each respondent's reported working country. In the collected responses, one respondent selected two of the countries of interest, and her/his response was examined as a response for both countries. Another respondent selected one of the aforementioned countries and one non-European country, and her/his response was examined as a response for the aforementioned country of interest. Responses that did not specify a specific country of work were excluded. Table 1 provides more information.

**Table 1:** The number of responses of each country and of each region

Region	Country	The number of valid responses by country	The number of valid responses by region
Nordic countries	Denmark	2	265
	Finland	42	
	Sweden	221	
Northwestern Europe	Belgium	72	444
	France	16	
	Germany	93	
	the Netherlands	64	
	Switzerland	66	
	the UK	133	
Southern Europe	Italy	56	101
	Spain	45	
Total			810

Because invalid answers were removed, the total number of responses to several demographic questions does not sum up to 810. Only responses with fewer than two invalid answers were considered valid and analyzed. Tables 2 provides the exact figures.

**Table 2:** Demographic characteristics of the respondents

Variables	Percentage of respondents (%)				P value^a^		
	Total	N	S	NW	N VS. S	N VS. NW	S VS. NW
*Age (n* = *808)*							
< = 30y	11.0	7.6	5.0	14.4	< 0.001	< 0.001	< 0.001
31-40y	32.6	36.5	10.9	35.4			
41-50y	22.0	20.2	22.8	23.0			
51-60y	21.5	18.2	34.6	20.5			
> 60y	12.7	17.5	26.7	6.8			
*Age (n* = *808)*	N	263	101	444	< 0.001	0.002	< 0.001
	Mean	46.1	52.8	42.8			
	Std	12.91	11.11	11.42			
*Gender (n* = *809)*							
Female	44.6	47.4	45.5	42.8			
Male	55.3	52.6	54.5	57.2			
*Mother tongue (n* = *810)*							
English	15.9	8.7	1.0	23.6	0.008	< 0.001	< 0.001
Other	84.1	91.3	99.0	76.4			
*Current academic position (n* = *810)*							
Professor	25.1	18.9	38.6	25.7	< 0.001	0.001	< 0.001
Associate professor	19.4	23.8	32.7	13.7			
Assistant professor	10.6	8.3	19.8	9.9			
Postdoc	24.7	27.6	3.0	27.9			
Other	18.4	17.7	5.9	21.6			
Not a scientific researcher	1.8	3.8	0	1.1			
*PhD degree (n* = *810)*							
Yes	82.7	88.3	80.2	80.0	0.005	0.016	
Current PhD candidate	8.9	6.8	5.0	11.0			
No	8.4	4.9	14.8	9.0			
*Year of obtaining PhD degree (n* = *643)*							
< 1979	2.8	3.5	8.0	1.2	< 0.001		< 0.001
1979–1988	7.6	6.2	14.7	7.0			
1989–1998	21.9	18.1	38.7	20.8			
1999–2008	28.1	29.5	22.7	28.4			
2009–2018	39.5	42.7	16.0	42.5			
*International research experience (*> *6 months) (n* = *810)*							
Yes	61.7	61.5	54.5	63.5			
No	38.3	38.5	45.5	36.5			

^a^P values based on Chi square tests of pairwise comparisons between the three regions. P values are only listed when P < 0.017

N, S and NW stand for Nordic countries (Denmark, Finland, and Sweden), Southern European countries (Italy and Spain), and Northwestern European countries (Belgium, France, Germany, the Netherlands, Switzerland, and the UK)

### Demographic characteristics

The demographic characteristics of the respondents are listed in Table 2. The age, mother tongue, and academic positions of respondents differed between regions, with differences being generally larger between southern Europe and the other two regions than between the latter two regions.

Respondents from southern Europe were over 50 years old on average (52.8 y, SD 11.1), i.e. significantly older than those from northern Europe (46.1 y, SD 12.9) and northwestern Europe (42.8 y, SD 11.4). Male respondents (55.3%) outnumbered female respondents, with no significant differences between the three regions. English was the mother tongue of a minority of respondents (15.9%) (8.7% in Northern Europe, 1.0% in southern Europe, and 23.6% in northwestern Europe, P < 0.05). Professors (25.1%), associate professors (19.4%), and postdoctoral researchers (24.7%) made up the majority of the respondents. In southern Europe, the proportion of senior researchers (professors 38.6%, associate professors 32.7%) was higher (P < 0.05) than in the other two regions. Four-fifths of those surveyed held a doctorate, the majority of which had been obtained since 1999 (67.6%). Around two-thirds of the respondents had more than six months of international research experience.

We conducted all logistic regression analyses correcting for age, mother tongue (English or not), current academic position and PhD degree to create adjusted odds ratios (aOR) with 95% confidence intervals (CI), taking into account demographic patterns in the three regions.

### Responses

#### Understanding of particular practices

**Fig. 1:**
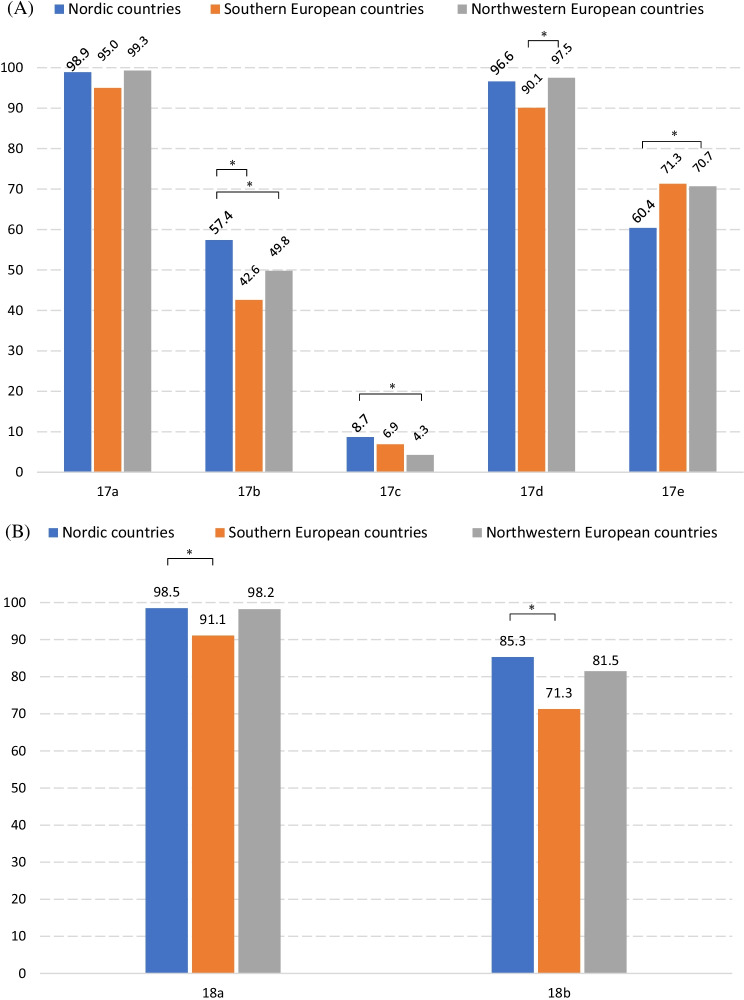

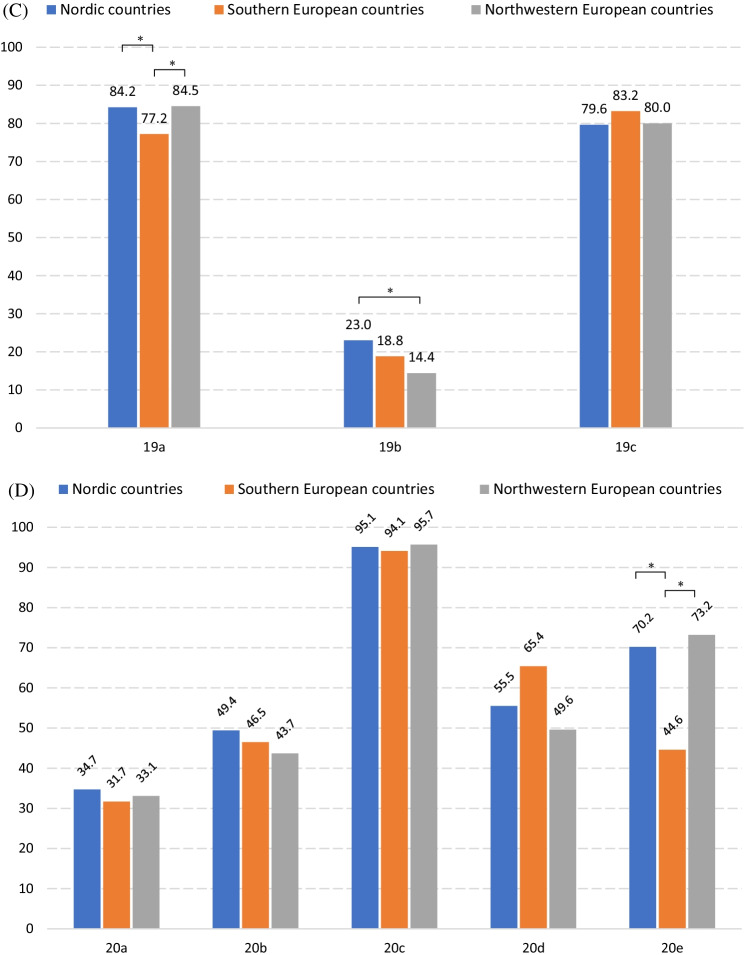
Percentage of respondents who regarded the practice as plagiarism. **A** Statement 17. Appropriation of others’ text, image and ideas. a. Copying text from someone else's publication without crediting the source. b. Copying text from someone else's publication with crediting the source, but without quotation marks. c. Copying text from someone else's publication with crediting the source and with quotation marks. d. Copying an image from someone else's publication without crediting the source. e. Using idea(s) from someone else's publication without crediting the source. **B** Statement 18. Appropriation of online sources a. Copying text from an online source without crediting the source. b. Copying text from an online source that has no list of authors, and without crediting the source. **C** Statement 19. Rephrasing or summarizing another person’s work a. Rephrasing another person’s work without crediting the source. b. Rephrasing text from someone else's publication without significant modification of the original, but with crediting the source. c. Summarizing another person’s work without crediting the source. **D** Statement 20. Text resources of article writing a. Paying someone else to write a paper without granting authorship. b. Having someone else to write a paper for free without granting authorship. c. Putting together pieces from different publications, and presenting the result as one’s own work. d. When writing a literature review, using the same framework of others’ review, without crediting the source. e. With permission from the original author, using another’s text without crediting the source. * There is significant difference after adjustments for  age, mother tongue, current academic position and PhD degree

In summary, the vast majority of responders (over 95% of the total) were successful in identifying the most evident types of plagiarism, including **Copying text from someone else's publication without crediting the source** (98.6%), **Copying an image from someone else's publication without crediting the source** (96.3%), **Copying text from an online source without crediting the source** (97.4%), **Putting together pieces from different publications, and presenting the result as one’s own work** (95.3%), **Republishing others’ work in another language without crediting the source** (98.4%). Compared to blatant plagiarism, such as copying and pasting text (without attribution), other practices appeared less obvious and were viewed as plagiarism by fewer respondents, such as **Rephrasing another person’s work without crediting the source** (83.4%), **Copying text from an online source that has no list of authors, and without crediting the source** (81.5%), **Using idea(s) from someone else's publication without crediting the source** (67.4%) and **Copying text from someone else's publication with crediting the source, but without quotation marks** (51.4%) (Fig. 1; Additional File 4: Table 1).

The aforementioned questions elicited generally similar responses from respondents in the three regions. However, in several circumstances, respondents from the three regions differed in their likelihood of not considering particular actions as plagiarism.

##### Northern Europe versus southern Europe

In comparison to respondents from southern Europe, more Nordic respondents viewed the following practices as plagiarism: **Copying text from someone else's publication with crediting the source, but without quotation marks** (aOR_N|S_ 1.80, 95% CI 1.10;2.95), **Copying text from an online source without crediting the source** (aOR_N|S_ 6.90, 95% CI 1.74;27.35), **Copying text from an online source that has no list of authors, and without crediting the source** (aOR_N|S_ 2.22, 95% CI 1.23;4.00), **Rephrasing another person’s work without crediting the source** (aOR_N|S_ 1.99, 95% CI 1.08;3.67), **With permission from the original author, using another’s text without crediting the source** (aOR_N|S_ 3.16, 95% CI 1.90;5.25). Only for one practice were Nordic respondents less likely than southern respondents to report the specific practice as plagiarism—**Reusing one’s own previously rejected research proposal for another funding application without crediting the source** (aOR_N|S_ 0.46, 95% CI 0.22;0.98) (Fig. 1; Additional File 4: Table 1).

##### Northern Europe versus northwestern Europe

In contrast to respondents from the northwest, Nordic respondents were more likely to identify the following practices as plagiarism: **Copying text from someone else's publication with crediting the source, but without quotation marks** (aOR_N|NW_ 1.55, 95% CI 1.12;2.16), **Copying text from someone else's publication with crediting the source and with quotation marks** (aOR_N|NW_ 2.20, 95% CI 1.12;4.31), **Rephrasing text from someone else's publication without significant modification of the original, but with crediting the source** (aOR_N|NW_ 2.06, 95% CI 1.35;3.12), **One has submitted work as dissertation/thesis, and submits parts of it to a journal afterwards without crediting the source** (aOR_N|NW_ 3.22, 95% CI 2.27;4.59), **One has submitted work as dissertation/thesis, and submits a summary of it to a journal afterwards without crediting the source** (aOR_N|NW_ 2.61, 95% CI 1.82;3.74). Only for one practice were Nordic respondents less likely than northwestern respondents to report the specific practice as plagiarism—**Using idea(s) from someone else's publication without crediting the source** (aOR_N|NW_ 0.64, 95% CI 0.46;0.90) (Fig. 1; Additional File 4: Table 1).

Figure 1 Percentage of respondents who regarded the practice as plagiarism

##### Southern Europe versus northwestern Europe

There were also differences between southern and northwestern Europe. Southern European respondents were less inclined than their northwestern European counterparts to identify certain acts as plagiarism, including **Copying an image from someone else's publication without crediting the source** (aOR_S|NW_ 0.29, 95% CI 0.10;0.82), **Rephrasing another person’s work without crediting the source** (aOR_S|NW_ 0.50, 95% CI 0.28;0.91), **With permission from the original author, using another’s text without crediting the source** (aOR_S|NW_ 0.26, 95% CI 0.16;0.42). Southern European respondents were, on the contrary, more likely to identify a few other behaviors as plagiarism than their counterparts in the northwest: **Reusing one’s own previously rejected research proposal for another funding application without crediting the source** (aOR_S|NW_ 2.40, 95% CI 1.20;4.80), **One has submitted work as dissertation/thesis, and submits parts of it to a journal afterwards without crediting the source** (aOR_S|NW_ 1.96, 95% CI 1.19;3.24), **One has submitted work as dissertation/thesis, and submits a summary of it to a journal afterwards without crediting the source** (aOR_S|NW_ 1.80, 95% CI 1.08;3.01) (Fig. 1; Additional File 4: Table 1).

#### Other perspectives on plagiarism

The survey examined more general perspectives on plagiarism in addition to understanding specific behaviors.

**Fig. 2:**
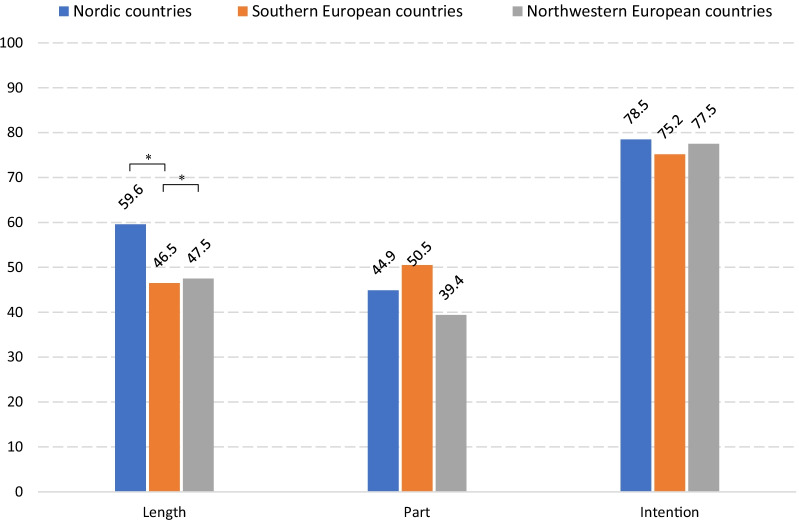
Percentage of respondents who selected each option to Question 15. Question 15: Which factor(s) do you think decide whether a body of copied and unattributed text constitutes plagiarism or not? a. The length of the copied text. b. The part of the copied text. c. The presence of an intention to copy without attribution. *There is significant difference after adjustments for age, mother tongue, current academic position and PhD degree

Some respondents believed that several variables were important in determining whether or not an act would be plagiarism. More precisely, 77.5%, 51.4%, and 42.6% of respondents, respectively, regarded **the intention**, **the length of the copied text**, **the part of the copied text** to be crucial. In contrast to respondents from southern and northwestern Europe, more Nordic respondents tended to believe that the length of copied text was important [aOR_N|S_: 1.76 (95% CI 1.08;2.87), aOR_N|NW_ 1.48 (95% CI 1.06;2.05)] (Fig. 2; Additional File 4: Table 2).

Figure 2 Percentage of respondents who selected each option to Question 15

Overall 34% reported to have been unsure whether they had been plagiarizing, but with Nordic respondents (26.8%) being less likely than northwestern respondents (39.6%) to **doubt whether they had been plagiarizing** (aOR_N|NW_: 0.61, 95% CI 0.43;0.86) (Additional File 4: Table 2).

**Fig. 3:**
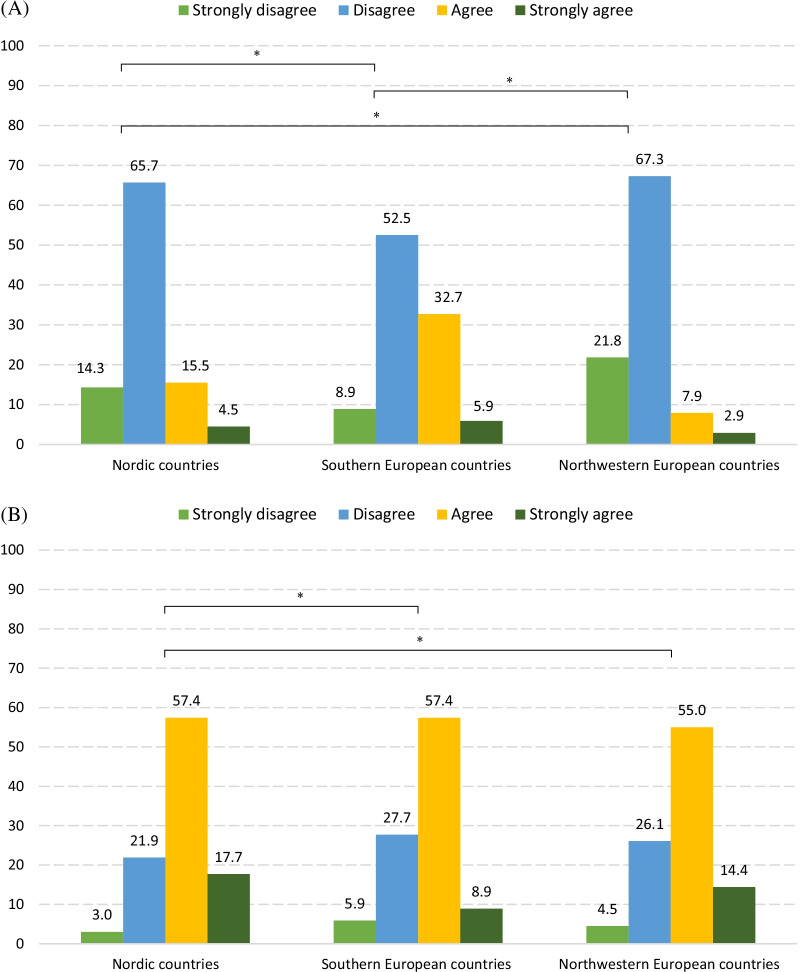

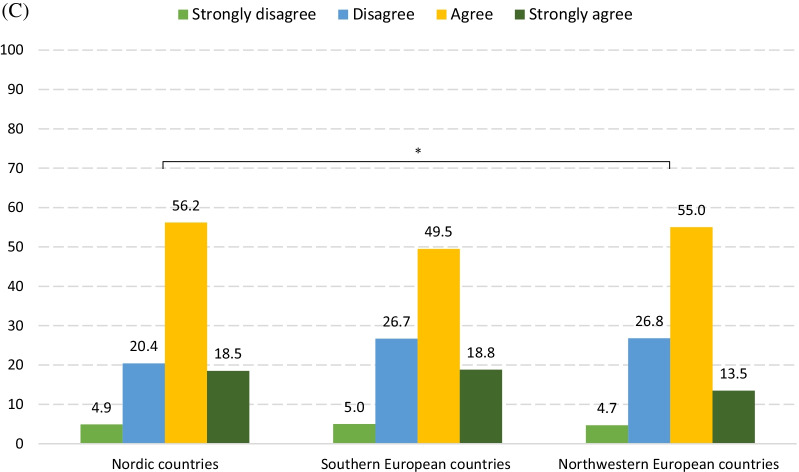
Percentage of respondents who selected each option to Question 12–14. **A** Attitudes to statement 12 “Plagiarism is a greater threat to biomedical research than data falsification”. **B** Attitudes to statement 13 “Plagiarism is a greater threat to biomedical research than granting co-authorship to someone whose contribution doesn’t justify it”. **C** Attitudes to statement 14 “Plagiarism is a greater threat to biomedical research than submitting a manuscript to more than one journals simultaneously”. * There is significant difference after adjustments for age, mother tongue, current academic position and PhD degree

In general, the majority of respondents agreed (or strongly agreed) that plagiarism was a higher threat to biomedical research than **﻿submitting a manuscript to more than one journal simultaneously** (70.5%) and ﻿**granting co-authorship to someone whose contribution doesn’t justify it** (70.8%), but a lesser hazard than **data falsification** (82.7%) (Fig. 3; Additional File 4: Table 3). When compared to **data falsificatio**n, respondents in the northwest disagreed the most that plagiarism constituted a higher threat to biomedical research, while those in the south agreed the most. When compared to **granting co-authorship to someone whose contribution doesn’t justify it**, Nordic respondents agreed the most that plagiarism constituted a higher threat to biomedical research. When comparing plagiarism to **submitting a manuscript to more than one journal simultaneously**, respondents from the north agreed more than respondents from the northwest that plagiarism was a higher concern.

Figure 3 Percentage of respondents who selected each option to Question 12-14

## Discussion

### Comparison of the three European regions

The initial goal of our survey was to explore biomedical professionals' general perceptions of plagiarism in Europe and China, as well as the similarities and contrasts between the two regions [14]. Like others [27–29], we focused initially on the similarities and differences of perceptions between the Western World and the Eastern World, and did not address potential disparities within Europe [14]. However, it was also apparent that the responses from European respondents exhibited geographical heterogeneity. Fortunately, the data we had gathered allowed for more in-depth research into perceptions of plagiarism across Europe. The present subsequent analysis provides additional novel empirical evidence about commonalities and disparities in the views held by biomedical researchers with regard to plagiarism.

Here, we did not repeat association analysis between responses and general demographic variables because this was covered in the 2020 article [14]. The purpose of the present analysis was to compare and contrast responses in three European regions: the Nordic countries, southern Europe, and northwestern Europe.

#### The main findings

Not unexpectedly, the main conclusions of the current analysis are very comparable to what we found in our 2020 article [14]. For example, in general, the perceived harm of plagiarism lies between that caused by data falsification and inappropriate authorship, multiple submission**.** Furthermore, the existence of intent was deemed more relevant than the part or length of the copied text in identifying a plagiarism act. A similar tendency was also observed in the present study about perceptions of specific practices: most respondents in the three European regions correctly identified the blatant plagiarism practices, but they identified the subtle ones less correctly. Nonetheless, even the most blatant forms of plagiarism, such as appropriation of another's text or image without PROPER attribution, which are clearly classified as plagiarism in many widely accepted guidelines [30–32], were never identified by 100% of the respondents.

Nevertheless, the proportions of respondents who did not identify/consider some specific practices as plagiarism varied across the three studied geographical regions within Europe. In other words, the comparison of the three regions reveals some intra-European divergent perspectives on plagiarism, despite showing broadly similar response patterns.

We need to state that we did not claim all of the listed practices in Sect. 2 of our questionnaire constituted plagiarism. By mixing plagiarism and non-plagiarism (undefined according to the current universal guidelines) practices, we wanted to investigate the respondents’ views of these practices. Among those practices, some universally constitute evident plagiarism, such as **Copying text from someone else’s publication without crediting the source**, and **Copying an image from someone else's publication without crediting the source**. Other practices might seem less straightforward to some, such as **Copying text from someone else's publication with crediting the source, but without quotation marks**, and **Rephrasing text from someone else's publication without significant modification of the original, but with crediting the source**. It is interesting to see how similarly or dissimilarly the respondents from the three European regions viewed these practices.

#### Summary of the comparison

According to our findings, more agreement was reached for the evident plagiarism practices (i.e. they were identified by over 90% of the respondents), and more divergences existed for the other practices, including the less evident plagiarism practices and those undefined practices.

In summary, compared with the other two European regions, Nordic respondents were more inclined to recognize the listed practices as plagiarism. Compared to the southern respondents, Nordic and northwestern respondents tended more frequently to consider less evident practices as plagiarism, such as Rephrasing another person’s work without crediting the source and With permission from the original author, using another’s text without crediting the source. In contrast, the southern respondents were the most likely to identify recycling of one’s previously rejected research proposal as plagiarism.

### Literature on perceptions of plagiarism across Europe

Earlier studies on plagiarism and research misconduct were generally conducted by researchers from English-speaking countries [7, 8, 33–36], and cultural factors were primarily focused on Western and Asian countries [34, 37]. Some recent research has begun to focus on intra-European differences, and some distinctions have been identified. The project IPPHEAE, whose conclusions have been documented in scientific articles and reports [37–42], is one of the most significant projects.

﻿The IPPHEAE project investigated higher education institutions (including students and staff) in 27 countries across Europe to see how they dealt with plagiarism and academic misconduct. Despite limited response rates in a few nations, the project yielded a wealth of data for cross-sectional comparison, even taking into account potential limitations in terms of representativeness.

When presenting the outcomes of the IPPHEAE project, Glendinning [37] noted “great variability in understanding what constitutes plagiarism and what was deemed acceptable academic practice” and pointed out that “the lack of consensus over what constitutes plagiarism is perhaps one of the major barriers to academic integrity across the EU.” Years later, in our survey, “the lack of consensus over what constitutes plagiarism” mentioned by the report has been documented again among the European biomedical researchers.

Although IPPHEA is a country-based research project, a general trend (without statistical analysis) is apparent from the IPPHEAE findings: the Nordic respondents (especially those in Finland and Sweden) were more likely to identify the two specific practices [(a) *﻿40% word-for-word copied work with no quotations,* (d) *40% copied work, ﻿with some words changed with no quotations, references or in text citations.*] as plagiarism than their counterparts in northwestern Europe (especially those in France, Germany, and the Netherlands), while the latter were more likely to do so than their counterparts in southern Europe (quantitative data is available from Spain and unavailable from Italy), which was generally consistent with their reported training experience [40, 41, 43–46].

A few more studies, in addition to IPPHEAE, also looked into perceptions of plagiarism across Europe, with or without providing detailed data.

Kayaoğlu et al. examined students' perceptions of plagiarism in three countries: Turkey, Germany, and Georgia, and discovered that German students were more sensitive to plagiarism and better at detecting it [9]. The disparity, according to Kayaoğlu et al. [9], is due to Turkey's "textbook-based" teaching strategy and exam-driven education system, as well as Georgia's similar cultural learning tradition with Asia.

Pupovac et al. studied four European nations and discovered that students in Bulgaria and Croatia were more tolerant of exam cheating than their counterparts in the UK [47]. They also expressed that their findings support Magnus’ conclusion [48] that tolerance for academic misbehavior was greater in post-communist countries.

Liaw et al. observed no significant correlation between nursing students’ self-confidence and clinical performance [49]. Similarly, the IPPHEAE project discovered that self-confidence was not necessarily favorably correlated with understanding or training of plagiarism [41, 43, 45]. Yaniv et al. [50] reported a dissociation between confidence and accuracy, whereby people tend to have confidence in consensus, even it is less accurate. As a consequence, it is possible that the respondents who reported to be confident with their research practices had experienced more consensus, regardless of how correct it was, on plagiarism definitions and practices. Besides, education and training experiences on the topic of plagiarism might lead those scientific researchers to assume that they had already developed a good understanding of it. In the present work, it has been observed that the Nordic respondents had lower degree of self-doubt of their research practices than their northwestern counterparts. Nevertheless, no difference was revealed between the other regions. The finding here that researchers’ self-confidence was not always positively associated to their knowledge was consistent with the above studies.

### Policy and training on research integrity across Europe

The various policies and training on research integrity across Europe might help us to understand the differences we observed between the three European regions.

Back in the year of 2013, ﻿Godecharle et al. observed disagreement across Europe in terms of national research integrity guidelines and research integrity training [51, 52]. In addition to the finding that ﻿the Nordic countries and most countries of central and western Europe have national guidelines, they also pointed out that two Nordic countries—Denmark and Norway—have a specific law to address research misconduct [52]. ﻿Resnik et al. also detected diversity after investigation into the﻿ national research misconduct policies of the TOP 40 ﻿research and development funding countries, around half of which were European countries [53]. Nevertheless, the intra-Europe consensus has not significantly improved by time. After examining the national regulatory documents on research integrity of ﻿32 countries of the European Free Trade Association in 2020, Desmond and Dierickx expressed worries that the ﻿ “core-periphery” model of harmonization has not yet been realized and that the divergences on national guidelines would pose threats to fairness and ﻿credibility when addressing research misconduct [54]. By reviewing research integrity training in ﻿11 of the 23 members of the League of European Research Universities (LERU), Abdi et al. also ﻿ found substantial variation across Europe and that educational resources mainly originated from northern and western Europe [55].

These limited comparative empirical studies have indicated that the northern (and western) European countries are more advanced in their guidelines and training on research integrity. Though the impact on attitudes and practices remain in doubt [56], education and training of research integrity are believed to improve an individual’s knowledge of research integrity and misconduct [57, 58]. Influences of guidelines also depend on training and education of research integrity. Accordingly, the Nordic respondents in our survey did show higher sensitivity to plagiarism-related/like practices. Compared to the southern respondents, the northern and northwestern respondents did show a more frequent ability to identify the less obvious plagiarism practices, such as inappropriate rephrasing. These findings further remind us of the necessity to promote harmonized approaches to research integrity policy and training in Europe.

### Comparison with China

Although we had already compared the European responses and Chinese responses in our original article [14], it was also of interest to compare the response patterns of each European region with that found for China.

After statistical analysis corrected for differences indemographic factors, some conclusions about discrepancies between Europe and China could be refined compared to our previous work [14]: for example, compared to the two or three European regions, the Chinese respondents were less likely to identify improper referencing as plagiarism (statement 17b, 17c). On the contrary, compared to the southern European respondents, the Chinese respondents were more likely to identify permitted unattributed text appropriation as plagiarism (statement 20e). Of the three European regions, the Nordic pattern of responses differed the most from the Chinese pattren, with the former being more likely to report a few specific practices as plagiarism (Additional files 5 and 6–1, 6–2, 6–3).

It is worth strssing that, as in our previous article, the main goal of our comparative analysis was to help understand different research behaviors, rather than making value judgements of researchers’ perceptions of plagiarism.

With increasing globalization of scientific communications, as in many other areas nowadays, researchers with different cultures and backgrounds are very likely to face the same assessment criteria of research practices. We suggest that understanding the differences is critical for understanding practical differences and addressing plagiarism more effectively.

### Limitations

There are limitations that we should be aware of when interpreting the findings.

One of the most typical biases in surveys on sensitive topics is response bias, and the respondents of our survey were researchers from leading universities, which can lead to the outcome being an estimation of “a better condition”. Besides, considering the low response rate (5.6%, of the European respondents) of our initial survey (possible reasons have been discussed in the previous study [14]), we should be aware that those answering the questionnaire might have a better understanding or higher English fluency than the others. In addition, the researchers from leading universities were highly exposed to the international research environment (around 60% of the respondents had more than 6 months’ international research experience), which might result in high homogeneity in terms of their understanding of plagiarism. It is conceivable that more discrepancies would have been observed if “less excellent” universities were included for comparison. Moreover, our sampling strategy led to the results of each region being more reflective of particular countries (and no data from central and eastern European countries), which might limit the study's representativeness. As a result, we should be cautious in extrapolating the findings.

Due to the length of the questionnaire (considering that too long questionnaires might decrease the response rate), some practices could not be described in many details, which might influence the respondents’ responses.

The Turnitin definition of plagiarism [18] and our previous work [19, 20] were used to design and improve our survey instrument. Although we had it improved by consulting experts and performing a trial survey, we nonetheless acknowledge that the instrument had not been formally validated.

The current study was based on replies gathered in 2018, which was more than three years ago. Given that people's perceptions regarding plagiarism may have shifted, especially in light of the increased public spotlight on research integrity and misconduct, it would be ideal if more up-to-date figures were accessible. To our knowledge, however, there are few prior studies that have sought to quantitatively analyze how biomedical scientists perceive plagiarism and compare responses across European nations, especially with such a large number of replies. As a result, we believe this research does still yield insightful and useful results.

### Practical implications

The empirical evidence in the present study has proved exitence of disagreement on what plagiarism is across Europe.

The first step towards harmonization of research integrity standards in Europe might be to reach an agreed and clear definition of research misconduct, including what is plagiarism and what it is not. Effective approaches of research integrity training, including education about plagiarism definitions is also needed.

## Conclusion

The present study has observed overall good understanding of plagiarism, particularly in its obvious forms, among European biomedical researchers. Although the three European areas have a comparable understanding of most practices, there are differences across them. In summary, the Nordic researchers identified the most types of practices as plagiarism. Nordic and northwestern respondents were more inclined than their southern counterparts to consider less evident practices as plagiarism, whereas the southern respondents were the most likely to recognize recycling of one’s previously rejected research proposal as plagiarism. When it comes to plagiarism and research misconduct, these similarities and differences throughout Europe should be taken into account.

## Supplementary Information

**Additional file 1**. Survey on Perceptions of Plagiarism Definition..

**Additional file 2**. Universities included in the survey

**Additional file 3**. Invitation to the online survey.

**Additional file 4**. Tables with details for comparison of the three European regions.

**Additional file 5.** Comparison between China and the three European regions.

**Additional file 6**. Comparison between the four regions (Nordic countries, southern European countries, northwestern European countries and China) for Statement 17-20.

**Additional file 7**. Comparison between the four regions (Nordic countries, southern European countries, northwestern European countries and China) for Question 15.

**Additional file 8**. Comparison between the four regions (Nordic countries, southern European countries, northwestern European countries and China) for Question 12-14.

## Data Availability

The datasets used during the current study are available from the corresponding author on reasonable request.

## Abbreviations

FFP: Fabrication, falsification and plagiarism
N: Nordic countries
NW: Northwestern European countries
S: Southern European countries
OR(s): Odds ratio(s)
aOR(s): Adjusted odds ratio(s)
CI(s): Confidence interval(s)
SD: Standard deviation
aOR_N|S_: Adjusted odds ratio of the northern Europe, with the southern Europe as the reference
aOR_N|NW_: Adjusted odds ratio of the northern Europe, with the northwestern Europe as the reference
aOR_S|NW_: Adjusted odds ratio of the southern Europe, with the northwestern Europe as the reference
