# Supplementary material for: Do biomedical researchers differ in their perceptions of plagiarism across Europe? Findings from an online survey among leading universities
Source: BMC Med Ethics. 2022 Aug 8;23:78. doi: 10.1186/s12910-022-00818-4 (PMC9358876; doi:10.1186/s12910-022-00818-4)
Supplement: Supplementary file 4 — Additional file 4. Tables with details for comparison of the three European regions. [file 12910_2022_818_MOESM4_ESM.docx]

**Additional file 4**

Tables with details for comparison of the three European regions

**Table 1** Percentage of respondents who regarded the practice as plagiarism

| Statement of practice | Percentage of respondents (%) | | | | P value^a^ | | | Adjusted OR (95% CI)^b^ | | |
| --- | --- | --- | --- | --- | --- | --- | --- | --- | --- | --- |
|  | Total (n=810) | N (n= 265) | S (n=101) | NW (n=444) | N vs. S | N vs. NW | S vs. NW | N vs. S | N vs. NW | S vs. NW |
| **Statement 17. Appropriation of others’ text, image and ideas** |  |  |  |  |  |  |  |  |  |  |
| a. Copying text from someone else's publication without crediting the source. | 98.6 | 98.9 | 95.0 | 99.3 | 0.026 |  | 0.001 | 2.92 (0.53;15.97) | 0.64 (0.11;3.70) | 0.22 (0.04;1.27) |
| b. Copying text from someone else's publication with crediting the source, but without quotation marks. | 51.4 | 57.4 | 42.6 | 49.8 | 0.011 |  |  | **1.80 (1.10;2.95)** | **1.55 (1.12;2.16)** | 0.86 (0.54;1.38) |
| c. Copying text from someone else's publication with crediting the source and with quotation marks. | 6.0 | 8.7 | 6.9 | 4.3 |  | 0.016 |  | 1.20 (0.46;3.13) | **2.20 (1.12;4.31)** | 1.83 (0.69;4.83) |
| d. Copying an image from someone else's publication without crediting the source. | 96.3 | 96.6 | 90.1 | 97.5 | 0.012 |  | <0.001 | 2.23 (0.75;6.62) | 0.64 (0.24;1.72) | **0.29 (0.10;0.82)** |
| e. Using idea(s) from someone else's publication without crediting the source. | 67.4 | 60.4 | 71.3 | 70.7 |  | 0.005 |  | 0.68 (0.40;1.15) | **0.64 (0.46;0.90)** | 0.94 (0.56;1.58) |
| **Statement 18. Appropriation of online sources** |  |  |  |  |  |  |  |  |  |  |
| a. Copying text from an online source without crediting the source. | 97.4 | 98.5 | 91.1 | 98.2 | <0.001 |  | <0.001 | **6.90 (1.74;27.35)** | 2.22 (0.56;8.79) | 0.32 (0.10;1.03) |
| b. Copying text from an online source that has no list of authors, and without crediting the source. | 81.5 | 85.3 | 71.3 | 81.5 | 0.002 |  | 0.021 | **2.22 (1.23;4.00)** | 1.41 (0.91;2.20) | 0.64 (0.37;1.10) |
| **Statement 19. Rephrasing or summarizing another person’s work** |  |  |  |  |  |  |  |  |  |  |
| a. Rephrasing another person’s work without crediting the source. | 83.4 | 84.2 | 77.2 | 84.5 |  |  |  | **1.99 (1.08;3.67)** | 1.00 (0.64;1.56) | **0.50 (0.28;0.91)** |
| b. Rephrasing text from someone else's publication without significant modification of the original, but with crediting the source. | 17.8 | 23.0 | 18.8 | 14.4 |  | 0.004 |  | 1.39 (0.75;2.57) | **2.06 (1.35;3.12)** | 1.48 (0.80;2.73) |
| c. Summarizing another person’s work without crediting the source. | 80.2 | 79.6 | 83.2 | 80.0 |  |  |  | 0.91 (0.48;1.70) | 0.92 (0.62;1.38) | 1.02 (0.55;1.88) |
| **Statement 20. Text resources of article writing** |  |  |  |  |  |  |  |  |  |  |
| a. Paying someone else to write a paper without granting authorship. | 33.4 | 34.7 | 31.7 | 33.1 |  |  |  | 1.13 (0.67;1.90) | 1.10 (0.78;1.56) | 0.98 (0.59;1.62) |
| b. Having someone else to write a paper for free without granting authorship. | 45.9 | 49.4 | 46.5 | 43.7 |  |  |  | 1.05 (0.64;1.70) | 1.28 (0.92;1.76) | 1.22 (0.76;1.95) |
| c. Putting together pieces from different publications, and presenting the result as one’s own work. | 95.3 | 95.1 | 94.1 | 95.7 |  |  |  | 2.02 (0.64;6.34) | 0.90 (0.42;1.97) | 0.45 (0.15;1.34) |
| d. When writing a literature review, using the same framework of others’ review, without crediting the source. | 53.4 | 55.5 | 65.4 | 49.6 |  |  | 0.004 | 0.70 (0.42;1.15) | 1.10 (0.79;1.52) | 1.58 (0.97;2.56) |
| e. With permission from the original author, using another’s text without crediting the source. | 68.6 | 70.2 | 44.6 | 73.2 | <0.001 |  | <0.001 | **3.16 (1.90;5.25)** | 0.81 (0.56;1.16) | **0.26 (0.16;0.42)** |
| **Statement 21. Publishing in multiple languages** |  |  |  |  |  |  |  |  |  |  |
| a. Republishing others’ work in another language without crediting the source. | 98.4 | 97.7 | 100.0 | 98.4 |  |  |  | 0.00 (0.00;32E238) | 0.82 (0.24;2.74) | 152132 (0.00;9E249) |
| b. Republishing one’s own work in another language without crediting the source. | 67.2 | 70.9 | 65.4 | 65.3 |  |  |  | 1.31 (0.78;2.21) | 1.27 (0.89;1.81) | 0.97 (0.59;1.59) |
| **Statement 22. Reuse of research proposal/publication** |  |  |  |  |  |  |  |  |  |  |
| a. Reusing one’s own previously rejected research proposal for another funding application without crediting the source. | 9.0 | 7.9 | 17.8 | 7.7 | 0.006 |  | 0.002 | **0.46 (0.22;0.98)** | 1.11 (0.61;2.04) | **2.40 (1.20;4.80)** |
| b. Reusing a significant portion of one’s own previous publication for a new publication without crediting the source. | 79.1 | 81.5 | 77.2 | 78.2 |  |  |  | 1.48 (0.82;2.69) | 1.26 (0.84;1.90) | 0.85 (0.48;1.51) |
| **Statement 23. Republication of dissertations** |  |  |  |  |  |  |  |  |  |  |
| a. One has submitted work as dissertation/thesis, and submits parts of it to a journal afterwards without crediting the source. | 32.3 | 47.2 | 37.6 | 22.3 | 0.100 | <0.001 | 0.001 | 1.65 (1.00;2.72) | **3.22 (2.27;4.59)** | **1.96 (1.19;3.24)** |
| b. One has submitted work as dissertation/thesis, and submits a summary of it to a journal afterwards without crediting the source. | 29.4 | 40.8 | 35.6 | 21.2 |  | <0.001 | 0.002 | 1.45 (0.87;2.42) | **2.61 (1.82;3.74)** | **1.80 (1.08;3.01)** |

^a^ P values based on Chi square tests of pairwise comparisons between the three regions. P values are only listed when P<0.05.

^b^ ORs (with 95% CIs) based on logistic regression analysis, with adjustments for age, mother tongue, current academic position and PhD degree. Reference is the second region.

N, S and NW stand for Nordic countries, Southern European countries and northwestern European countries.

**Table 2** Percentage of respondents who selected each option (Question 15-16)

| Question | Percentage of respondents (%, n=810) | | | | P value^a^ | | | | Adjusted OR (95% CI)^b^ | | | |
| --- | --- | --- | --- | --- | --- | --- | --- | --- | --- | --- | --- | --- |
|  | Total | N | S | NW | | N vs. S | N vs. NW | S vs. NW | | N vs. S | N vs. NW | S vs. NW |
| **Question 15. Which factor(s) do you think decide whether a body of copied and unattributed text constitutes plagiarism or not?** |  |  |  |  | |  |  |  | |  |  |  |
| a. The length of the copied text | 51.4 | 59.6 | 46.5 | 47.5 | | 0.024 | 0.002 |  | | **1.76 (1.08;2.87)** | **1.48 (1.06;2.05)** | 0.84 (0.52;1.34) |
| b. The part of the copied text | 42.6 | 44.9 | 50.5 | 39.4 | |  |  | 0.041 | | 0.84 (0.52;1.36) | 1.08 (0.78;1.50) | 1.29 (0.81;2.07) |
| c. The presence of an intention to copy without attribution | 77.5 | 78.5 | 75.2 | 77.5 | |  |  |  | | 1.08 (0.61;1.90) | 1.21 (0.81;1.79) | 1.12 (0.65;1.93) |
| **Question 16. Have you ever been unsure whether you are plagiarizing?** |  |  |  |  | |  |  |  | |  |  |  |
| a. Yes | 34.0 | 26.8 | 27.7 | 39.6 | |  | <0.001 | 0.026 | | 0.86 (0.50;1.48) | **0.61 (0.43;0.86)** | 0.70 (0.42;1.18) |

^a^ P values based on Chi square tests of pairwise comparisons between the three regions. P values are only listed when P<0.05.

^b^ ORs (with 95% CIs) based on logistic regression analysis, with adjustments for age, mother tongue, current academic position and PhD degree. Reference is the second region.

N, S and NW stand for Nordic countries, Southern European countries and northwestern European countries.

**Table 3** Percentage of respondents who selected each option (Question 12-14)

| Question | Percentage of respondents (%, n=810) | | | | P value^a^ | | | | Adjusted OR (95% CI)^b^ | | | |
| --- | --- | --- | --- | --- | --- | --- | --- | --- | --- | --- | --- | --- |
|  | Total | N | S | NW | | N vs. S | N vs. NW | S vs. NW | | N vs. S | N vs. NW | S vs. NW |
| **Q12. Greater threat than data falsification** |  |  |  |  | | 0.002 | 0.002 | <0.001 | | **0.49 (0.30;0.80)** | **1.68 (1.20;2.36)** | **3.43 (2.12;5.54)** |
| Strongly disagree | 17.8 | 14.3 | 8.9 | 21.8 | |  |  |  | |  |  |  |
| Disagree | 64.9 | 65.7 | 52.5 | 67.3 | |  |  |  | |  |  |  |
| Agree | 13.4 | 15.5 | 32.7 | 7.9 | |  |  |  | |  |  |  |
| Strongly agree | 3.8 | 4.5 | 5.9 | 2.9 | |  |  |  | |  |  |  |
| **Q13. Greater threat than granting co-authorship** |  |  |  |  | |  |  |  | | **1.64 (1.03;2.61)** | **1.42 (1.04;1.94)** | 0.87 (0.55;1.35) |
| Strongly disagree | 4.2 | 3.0 | 5.9 | 4.5 | |  |  |  | |  |  |  |
| Disagree | 24.9 | 21.9 | 27.7 | 26.1 | |  |  |  | |  |  |  |
| Agree | 56.0 | 57.4 | 57.4 | 55.0 | |  |  |  | |  |  |  |
| Strongly agree | 14.8 | 17.7 | 8.9 | 14.4 | |  |  |  | |  |  |  |
| **Q14. Greater threat than submitting to more than one journal** |  |  |  |  | |  |  |  | | 1.04 (0.66;1.66) | **1.47 (1.07;2.00)** | 1.40 (0.90;2.20) |
| Strongly disagree | 4.8 | 4.9 | 5.0 | 4.7 | |  |  |  | |  |  |  |
| Disagree | 24.7 | 20.4 | 26.7 | 26.8 | |  |  |  | |  |  |  |
| Agree | 54.7 | 56.2 | 49.5 | 55.0 | |  |  |  | |  |  |  |
| Strongly agree | 15.8 | 18.5 | 18.8 | 13.5 | |  |  |  | |  |  |  |

^a^ P values based on Chi square tests of pairwise comparisons between the three regions. P values are only listed when P<0.05.

^b^ ORs (with 95% CIs) based on logistic regression analysis, with adjustments for age, mother tongue, current academic position and PhD degree. Reference is the second region.

N, S and NW stand for Nordic countries, Southern European countries and northwestern European countries.
