# Supplementary material for: Do biomedical researchers differ in their perceptions of plagiarism across Europe? Findings from an online survey among leading universities
Source: BMC Med Ethics. 2022 Aug 8;23:78. doi: 10.1186/s12910-022-00818-4 (PMC9358876; doi:10.1186/s12910-022-00818-4)
Supplement: Supplementary file 5 — Additional file 5. Comparison between China and the three European regions. [file 12910_2022_818_MOESM5_ESM.docx]

**Additional 5 Comparison between China and the three European regions**

**Table 1** Percentage of respondents who regarded the practice as plagiarism

| Statement of practice | Percentage of respondents (%) | | | | Adjusted OR (95% CI) | | |
| --- | --- | --- | --- | --- | --- | --- | --- |
|  | China (n=204) | N (n= 265) | S (n=101) | NW (n=444) | China vs. N | China vs. S | China vs. NW |
| **Statement 17. Appropriation of others’ text, image and ideas** |  |  |  |  |  |  |  |
| a. Copying text from someone else's publication without crediting the source. | 93.6 | 98.9 | 95.0 | 99.3 | 0.43 (0.05;3.98) | 2.69 (0.23;31.22) | 0.306 (0.03;3.52) |
| b. Copying text from someone else's publication with crediting the source, but without quotation marks. | 36.3 | 57.4 | 42.6 | 49.8 | **0.23 (0.10;0.54)** | 0.40 (0.16;1.03) | **0.36 (0.15;0.84)** |
| c. Copying text from someone else's publication with crediting the source and with quotation marks. | 5.9 | 8.7 | 6.9 | 4.3 | **0.13 (0.04;0.45)** | **0.14 (0.03;0.62)** | **0.25 (0.07;0.91)** |
| d. Copying an image from someone else's publication without crediting the source. | 94.6 | 96.6 | 90.1 | 97.5 | 0.96 (0.14;6.54) | 2.62 (0.34;20.00) | 0.66 (0.09;4.66) |
| e. Using idea(s) from someone else's publication without crediting the source. | 65.7 | 60.4 | 71.3 | 70.7 | 0.76 (0.32;1.84) | 0.53 (0.20;1.39) | 0.46 (0.19;1.11) |
| **Statement 18. Appropriation of online sources** |  |  |  |  |  |  |  |
| a. Copying text from an online source without crediting the source. | 88.2 | 98.5 | 91.1 | 98.2 | 0.30 (0.04;2.18) | 1.87 (0.24;14.46) | 0.44 (0.06;3.18) |
| b. Copying text from an online source that has no list of authors, and without crediting the source. | 69.1 | 85.3 | 71.3 | 81.5 | **0.35 (0.12;0.98)** | 0.79 (0.26;2.41) | 0.49 (0.18;1.38) |
| **Statement 19. Rephrasing or summarizing another person’s work** |  |  |  |  |  |  |  |
| a. Rephrasing another person’s work without crediting the source. | 87.8 | 84.2 | 77.2 | 84.5 | 0.53 (0.15;1.82) | 0.99 (0.27;3.66) | 0.50 (0.15;1.71) |
| b. Rephrasing text from someone else's publication without significant modification of the original, but with crediting the source. | 13.7 | 23.0 | 18.8 | 14.4 | 0.40 (0.14;1.12) | 0.52 (0.16;1.67) | 0.77 (0.27;2.22) |
| c. Summarizing another person’s work without crediting the source. | 71.1 | 79.6 | 83.2 | 80.0 | 1.34 (0.54;3.32) | 1.27 (0.44;3.62) | 1.23 (0.49;3.08) |
| **Statement 20. Text resources of article writing** |  |  |  |  |  |  |  |
| a. Paying someone else to write a paper without granting authorship. | 52.4 | 34.7 | 31.7 | 33.1 | 0.79 (0.34;1.87) | 0.83 (0.32;2.16) | 0.83 (0.35;1.96) |
| b. Having someone else to write a paper for free without granting authorship. | 63.7 | 49.4 | 46.5 | 43.7 | 0.84 (0.36;1.96) | 0.88 (0.35;2.23) | 1.03 (0.44;2.41) |
| c. Putting together pieces from different publications, and presenting the result as one’s own work. | 91.7 | 95.1 | 94.1 | 95.7 | 0.45 (0.08;2.42) | 0.76 (0.12;4.99) | 0.40 (0.07;2.20) |
| d. When writing a literature review, using the same framework of others’ review, without crediting the source. | 53.4 | 55.5 | 65.4 | 49.6 | 0.67 (0.29;1.52) | 0.47 (0.19;1.17) | 0.72 (0.32;1.65) |
| e. With permission from the original author, using another’s text without crediting the source. | 61.3 | 70.2 | 44.6 | 73.2 | 0.87 (0.37;2.04) | **2.79 (1.10;7.09)** | 0.71 (0.30;1.66) |
| **Statement 21. Publishing in multiple languages** |  |  |  |  |  |  |  |
| a. Republishing others’ work in another language without crediting the source. | 98.5 | 97.7 | 100.0 | 98.4 | 3.57 (0.32;40.43) | 0.00 (0.00;62E239) | 3.14 (0.26;37.89) |
| b. Republishing one’s own work in another language without crediting the source. | 50.0 | 70.9 | 65.4 | 65.3 | **0.28 (0.12;0.68)** | **0.34 (0.13;0.91)** | **0.34 (0.14;0.83)** |
| **Statement 22. Reuse of research proposal/publication** |  |  |  |  |  |  |  |
| a. Reusing one’s own previously rejected research proposal for another funding application without crediting the source. | 18.6 | 7.9 | 17.8 | 7.7 | 0.76 (0.25;2.36) | 0.30 (0.08;1.05) | 0.75 (0.24;2.40) |
| b. Reusing a significant portion of one’s own previous publication for a new publication without crediting the source. | 77.4 | 81.5 | 77.2 | 78.2 | **0.24 (0.08;0.74)** | 0.33 (0.10;1.10) | **0.29 (0.10;0.89)** |
| **Statement 23. Republication of dissertations** |  |  |  |  |  |  |  |
| a. One has submitted work as dissertation/thesis, and submits parts of it to a journal afterwards without crediting the source. | 16.2 | 47.2 | 37.6 | 22.3 | **0.13 (0.05;0.32)** | **0.20 (0.07;0.55)** | 0.40 (0.15;1.03) |
| b. One has submitted work as dissertation/thesis, and submits a summary of it to a journal afterwards without crediting the source. | 13.2 | 40.8 | 35.6 | 21.2 | **0.33 (0.12;0.87)** | 0.47 (0.16;1.34) | 0.87 (0.32;2.32) |

ORs (with 95% CIs) based on logistic regression analysis, with adjustments for age, mother tongue, current academic position and PhD degree. Reference is the second region.

N, S and NW stand for Nordic countries, Southern European countries and northwestern European countries.

**Table 2** Percentage of respondents who selected each option (Question 15-16)

| Statement of practice | Percentage of respondents (%) | | | | Adjusted OR (95% CI) | | |
| --- | --- | --- | --- | --- | --- | --- | --- |
|  | China (n=204) | N (n= 265) | S (n=101) | NW (n=444) | China vs. N | China vs. S | China vs. NW |
| **Question 15. Which factor(s) do you think decide whether a body of copied and unattributed text constitutes plagiarism or not?** |  |  |  |  |  |  |  |
| a. The length of the copied text | 59.8 | 59.6 | 46.5 | 47.5 | 0.57 (0.25;1.32) | 0.96 (0.38;2.40) | 0.81 (0.35;1.88) |
| b. The part of the copied text | 55.4 | 44.9 | 50.5 | 39.4 | 0.75 (0.32;1.76) | 0.60 (0.24;1.53) | 0.78 (0.33;1.82) |
| c. The presence of an intention to copy without attribution | 67.2 | 78.5 | 75.2 | 77.5 | 0.82 (0.33;2.06) | 0.95 (0.34;2.62) | 1.02 (0.41;2.58) |
| **Question 16. Have you ever been unsure whether you are plagiarizing?** |  |  |  |  |  |  |  |
| a. Yes | 18.1 | 26.8 | 27.7 | 39.6 | 1.15 (0.44;3.03) | 0.98 (0.34;2.82) | 0.69 (0.26;1.80) |

ORs (with 95% CIs) based on logistic regression analysis, with adjustments for age, mother tongue, current academic position and PhD degree. Reference is the second region.

N, S and NW stand for Nordic countries, Southern European countries and northwestern European countries.

**Table 3** Percentage of respondents who selected each option (Question 12-14)

| Statement of practice | Percentage of respondents (%) | | | | Adjusted OR (95% CI) | | |
| --- | --- | --- | --- | --- | --- | --- | --- |
|  | China (n=204) | N (n= 265) | S (n=101) | NW (n=444) | China vs. N | China vs. S | China vs. NW |
| **Q12. Greater threat than data falsification** |  |  |  |  | 0.67 (0.31;1.45) | **0.31 (0.13;0.74)** | 1.06 (0.49;2.31) |
| Strongly disagree | 5.4 | 14.3 | 8.9 | 21.8 |  |  |  |
| Disagree | 47.6 | 65.7 | 52.5 | 67.3 |  |  |  |
| Agree | 30.9 | 15.5 | 32.7 | 7.9 |  |  |  |
| Strongly agree | 16.2 | 4.5 | 5.9 | 2.9 |  |  |  |
| **Q13. Greater threat than granting co-authorship to someone whose contribution doesn’t justify it** |  |  |  |  | 0.85 (0.39;1.85) | 1.30 (0.55;3.09) | 1.15 (0.52;2.51) |
| Strongly disagree | 2.4 | 3.0 | 5.9 | 4.5 |  |  |  |
| Disagree | 8.3 | 21.9 | 27.7 | 26.1 |  |  |  |
| Agree | 47.1 | 57.4 | 57.4 | 55.0 |  |  |  |
| Strongly agree | 42.2 | 17.7 | 8.9 | 14.4 |  |  |  |
| **Q14. Greater threat than submitting to more than one journals simultaneously.** |  |  |  |  | 0.58 (0.27;1.26) | 0.57 (0.24;1.35) | 0.81 (0.37;1.76) |
| Strongly disagree | 4.9 | 4.9 | 5.0 | 4.7 |  |  |  |
| Disagree | 11.8 | 20.4 | 26.7 | 26.8 |  |  |  |
| Agree | 52.9 | 56.2 | 49.5 | 55.0 |  |  |  |
| Strongly agree | 30.4 | 18.5 | 18.8 | 13.5 |  |  |  |

ORs (with 95% CIs) based on logistic regression analysis, with adjustments for age, mother tongue, current academic position and PhD degree. Reference is the second region.

N, S and NW stand for Nordic countries, Southern European countries and northwestern European countries.
