# Supplementary material for: Do biomedical researchers differ in their perceptions of plagiarism across Europe? Findings from an online survey among leading universities
Source: BMC Med Ethics. 2022 Aug 8;23:78. doi: 10.1186/s12910-022-00818-4 (PMC9358876; doi:10.1186/s12910-022-00818-4)
Supplement: Supplementary file 6 — Additional file 6. Comparison between the four regions (Nordic countries, southern European countries, northwestern European countries and China) for Statement 17-20. [file 12910_2022_818_MOESM6_ESM.docx]

**Additional file 6-1**

Comparison between the four regions (Nordic countries, southern European countries, northwestern European countries and China)

*

*

*

*

*

*

*

*

*

*

(1)

*

*

81.5

98.2

*

(2)

*

*

*

(3)

*

*

*

(4)

Figure 1. Percentage of respondents who regarded the practice as plagiarism

(1) Statement 17. Appropriation of others’ text, image and ideas

a. Copying text from someone else's publication without crediting the source.

b. Copying text from someone else's publication with crediting the source, but without quotation marks.

c. Copying text from someone else's publication with crediting the source and with quotation marks.

d. Copying an image from someone else's publication without crediting the source.

e. Using idea(s) from someone else's publication without crediting the source.

(2) Statement 18. Appropriation of online sources

a. Copying text from an online source without crediting the source.

b. Copying text from an online source that has no list of authors, and without crediting the source.

(3) Statement 19. Rephrasing or summarizing another person’s work

a. Rephrasing another person’s work without crediting the source.

b. Rephrasing text from someone else's publication without significant modification of the original, but with crediting the source.

c. Summarizing another person’s work without crediting the source.

(4) Statement 20. Text resources of article writing

a. Paying someone else to write a paper without granting authorship.

b. Having someone else to write a paper for free without granting authorship.

c. Putting together pieces from different publications, and presenting the result as one’s own work.

d. When writing a literature review, using the same framework of others’ review, without crediting the source.

e. With permission from the original author, using another’s text without crediting the source.

* There is significant difference after correction for age, mother tongue, current academic position and PhD degree differences.
