# Supplementary material for: Do biomedical researchers differ in their perceptions of plagiarism across Europe? Findings from an online survey among leading universities
Source: BMC Med Ethics. 2022 Aug 8;23:78. doi: 10.1186/s12910-022-00818-4 (PMC9358876; doi:10.1186/s12910-022-00818-4)
Supplement: Supplementary file 7 — Additional file 7. Comparison between the four regions (Nordic countries, southern European countries, northwestern European countries and China) for Question 15. [file 12910_2022_818_MOESM7_ESM.docx]

**Additional file 6-2**

Comparison between the four regions (Nordic countries, southern European countries, northwestern European countries and China)

*

*

Figure 2. Percentage of respondents who selected each option to Question 15:

**Which factor(s) do you think decide whether a body of copied and unattributed text constitutes plagiarism or not?**

a. The length of the copied text

b. The part of the copied text

c. The presence of an intention to copy without attribution

* There is significant difference after correction for age, mother tongue, current academic position and PhD degree differences.
