# Supplementary material for: Do biomedical researchers differ in their perceptions of plagiarism across Europe? Findings from an online survey among leading universities
Source: BMC Med Ethics. 2022 Aug 8;23:78. doi: 10.1186/s12910-022-00818-4 (PMC9358876; doi:10.1186/s12910-022-00818-4)
Supplement: Supplementary file 8 — Additional file 8. Comparison between the four regions (Nordic countries, southern European countries, northwestern European countries and China) for Question 12-14. [file 12910_2022_818_MOESM8_ESM.docx]

**Additional file 6-3**

Comparison between the four regions (Nordic countries, southern European countries, northwestern European countries and China)

*

*

*

*

(1)

*

*

(2)

*

(3)

Figure 3 Percentage of respondents who selected each option to Question 12-14:

(1) Attitudes to statement 12 “**Plagiarism** is a greater threat to biomedical research than **data falsification**”.

(2) Attitudes to statement 13 “**Plagiarism** is a greater threat to biomedical research than **granting co-authorship to someone whose contribution doesn’t justify it**”.

(3) Attitudes to statement 14 “**Plagiarism** is a greater threat to biomedical research than **submitting a manuscript to more than one journals simultaneously**”.

* There is significant difference after correction for age, mother tongue, current academic position and PhD degree differences.
